# Pervasive Undisclosed Conflicts of Interest in Applied Behavior Analysis Autism Literature

**DOI:** 10.3389/fpsyg.2021.676303

**Published:** 2021-05-05

**Authors:** Kristen Bottema-Beutel, Shannon Crowley

**Affiliations:** Lynch School of Education and Human Development, Boston College, Chestnut Hill, MA, United States

**Keywords:** autism, intervention – behavioral, applied behavior analysis, conflicts of interest, researcher ethics

## Abstract

Many autistic people (including researchers and non-researchers) are becoming increasingly involved in, and increasingly critical of, autism intervention research. They have expressed concerns regarding applied behavior analysis (ABA) interventions on a number of grounds, one of which is the prevalence of conflicts of interests (COIs) among autism intervention researchers. These concerns are now also being addressed by non-autistic researchers. COIs can introduce bias into the research process, and allow researchers to demonstrate positive effects for interventions that are not actually effective. Despite these concerns, there are no studies to date that examine the prevalence of COIs in behavioral journals. Because ABA services are routinely provided to autistic people in the United States as a means to address difficulties experienced by autistic people, this is an important area of investigation. We tallied author COIs in articles published over a 1-year period that tested, commented on, or reviewed ABA autism intervention strategies, extracted from eight journals devoted to publishing behavioral research. We coded included studies for COIs related to researcher employment as an ABA clinical provider or a training consultant to ABA clinical providers. We found that 84% of studies had at least one author with this type of COI, but they were only disclosed as COIs in 2% of studies. Additionally, 87% of studies with statements claiming the authors did not have COIs, were authored by researchers found to have clinical/training consultancy COIs. Pervasive, undisclosed COIs likely lead to researcher bias, and could at least partially account for persistent poor quality research in this area. The high prevalence of COIs among this research corroborates the concerns expressed by many autistic people. The autism community – including autistic people, autism researchers, and other stakeholders – should be aware of the prevalence of undisclosed COIs in this literature and take this into account when using, providing, or recommending ABA services.

## Introduction

In intervention research, conflicts of interests (COIs) occur when researchers can potentially benefit from demonstrating that interventions are effective in achieving particular outcomes (Gorman, 2018). Researcher COIs do not always indicate that a given study is biased, but failure to acknowledge COIs can mean that the researchers have not taken appropriate precautions to protect against the bias that COIs potentially introduce. In order to alert stakeholder communities to the presence of COIs that could introduce bias into the research process, most journals that publish intervention research instruct authors to disclose actual, potential, or perceived COIs. The Committee on Publication Ethics (COPE) was established in 1997 in an effort to improve research integrity, with COIs being a chief concern (Committee on Publication Ethics, 2019). Major contributions of COPE are disseminating guidance on the establishment of COI policies and offering procedural advice on resolving COI disputes to member journal editors.

Recently, a review of 150 group design intervention studies for young autistic children concluded that COIs are likely pervasive, but under-reported in this literature, despite the ubiquity of COI disclosure requirements (Bottema-Beutel et al., 2020). A limitation of this study is that it excluded single case designs (SCDs), which is the study design used by the majority of research into the effects of applied behavior analysis (ABA) interventions for autistic people (Dawson and Fletcher-Watson, 2020; described in detail below). It is important to explore COIs in this area of research because many autistic adults (including researchers and non-researchers) and non-autistic researchers have expressed serious ethical concerns about the provision of ABA to autistic people (Dawson, 2004; Devita-Raeburn, 2016), including concerns related to undisclosed COIs (Dawson, 2020). Autistic people are increasingly setting the agenda for autism research, and this includes critiques of intervention practices they may have received as children, or continue to receive into adulthood. Further, ABA interventions are routinely recommended by primary care providers in the United States to parents seeking support for their autistic children (Zwaigenbaum et al., 2015; Hyman et al., 2020), with more than 60% of autistic children in the receiving some form of behavioral intervention rooted in ABA philosophy (Xu et al., 2019). As such, it is important to determine if these recommendations are consistent with available literature and not unduly influenced by researcher bias.

Conflicts of interests vary in the extent to which they present clear-cut opportunities for researcher gain, and therefore also likely vary in the magnitude of their influence on researcher conduct. For example, COIs directly involving the researcher’s employment may be a larger source of bias than COIs that provide opportunities for prestige (which is arguably present in most intervention studies given publishing incentives that reward positive findings), but are not explicitly linked to financial gain. In the current study, we focus on the former type of COI; specifically on instances where the intervention researcher is also an ABA clinical provider and/or provides paid training consultation to ABA clinical providers. Employment related COIs are widely recognized as COIs that can contribute to researcher bias, and because of this are regularly required in journal submission policies to be disclosed in research reports.

### Applied Behavior Analysis

Applied behavior analysis is an approach to studying and modifying behavior that is based on the principles of behaviorism. Behaviorism is a theory of learning that asserts all behavior is learned via contingencies between antecedents (events preceding the production of a behavior), the behavior, and the consequences following the behavior (Watson, 1924/2017). Behavior that is followed by favorable outcomes will continue to occur, and behavior that is not followed by favorable outcomes will disappear from one’s repertoire (Roane et al., 2016). According to this theory, these contingencies can be leveraged to teach children and adults new behavior that expands upon or replaces existing behavior patterns. Principles of ABA were first formulated as an intervention program for autistic children in the early 1960s (Ferster and DeMyer, 1962) and later broadened into a more intensive program by Ivar Lovaas in the 1970s and 1980s (Lovaas, 1987). Since then, ABA services for autistic people have become widely available, and are provided in clinics, schools, and hospitals in the United States and internationally.

Many ABA proponents assert that ABA is not a single intervention approach but a variety of approaches that share underlying principles in regards to behavior and learning (Baer et al., 1968). However, the procedures used by ABA practitioners who provide services to autistic people are often marketed using the umbrella acronym “ABA,” even if those services vary in terms of intensity, focus, and delivery context. ABA interventions for autistic people are the most widely known and researched form of ABA intervention, and comprise a sub-specialty of certification by the Behavior Analysis Certification Board (2020). In most states in the US, professionals who provide ABA services are required to receive specialized training to become a Board Certified Behavior Analyst (BCBA), or be supervised by a BCBA. While some literature of reviews have concluded that behavioral approaches are efficacious for supporting autistic children (e.g., Zwaigenbaum et al., 2015), meta-analytic studies that have sufficiently examined study quality using well-established quality indicators (e.g., the Cochrane risk of bias tool; Higgins et al., 2011) report that there is insufficient evidence for these claims (e.g., Sandbank et al., 2020).

### Sources of Bias in Single Case Design Intervention Research

A behaviorist approach to understanding human behavior and learning invites the operationalization of discrete behaviors, to determine how they change after alteration of antecedents or consequences. Therefore, ABA intervention researchers often make use of SCDs, in which behaviors are observed and repeatedly measured prior to an intervention (i.e., the baseline condition) and again during the implementation of an intervention (i.e., the “treatment” condition). Through various techniques of staggering the onset of intervention procedures across participants, environments, and/or time, researchers can make claims about functional relations between the intervention procedures and changes in children’s observed behavior.

Unlike for group design research, there is no widely agreed upon tool for assessing bias in SCD studies. However, Reichow et al. (2018) propose that risks of bias in SCDs are analogous to risks of bias in group-design studies. They describe three risk of bias categories, including: (a) selection bias (systematic differences in baseline characteristics of participants), (b) performance bias (systematic differences between participants in care or exposure to factors other than the intervention), (c) and detection bias (systematic differences between participants in the measurement and reporting of outcomes). Each of these sources of bias can increase the likelihood that an intervention procedure will be determined to bear a functional relation with the outcome, when it in fact does not. For example, a researcher could assign participants to a control condition if they have some reason to suspect the intervention will not be successful for that student during a particular session (selection bias). Or, researchers may know which participants are assigned to an intervention condition, and treat them more favorably than participants in the control condition in ways unrelated to the intervention being examined (performance bias). Finally, researchers who track data on participant outcomes may be aware of when the child is in a treatment condition, and may score that child more favorably than when the child is in the control condition (detection bias). In addition to these sources of bias, researchers can also interpret evidence more favorably than is warranted, and determine that a set of intervention practices are effective for improving outcomes, when the data in fact do not support this assertion (Bottema-Beutel and Crowley, 2020).

Because risk of bias evaluation tools for SCDs are relatively new, there are only a few studies to date that have used them to evaluate ABA interventions. One recent review is notable, however. Davis et al. (2019) systematically evaluated research on non-pharmacological interventions for autistic adults over a 50 year period. The majority of included studies were SCDs examining ABA intervention techniques. Using Reichow’s risk of bias tool, they found that nearly 75% of included studies had a high risk of bias across all four domains described above. Bias in this area of study therefore appears widespread, and limits our ability to rely on evidence used to make claims of effectiveness. It is possible that author COIs contribute to the persistently large percentage of ABA studies that are low quality designs (see also Sandbank et al., 2020, for a similar evaluation of group design autism intervention research).

### The Current Study

In this study, we examined author COIs from articles focusing on interventions for autistic people, extracted from eight peer reviewed journals devoted to publishing research on ABA strategies. We selected ABA journals that represented a variety of publishers, and a range of impact factors. We also ensured that the top journals in the field were represented in our sample; the *Journal of Applied Behavior Analysis* is considered a flagship ABA journal (Kranak et al., 2020), and *Behavior Modification* has a similar Impact Factor in the year this study was conducted. Our aims were to determine: (a) the proportion of articles with one or more authors who either provided ABA clinical services or provided private training to ABA practitioners, (b) the proportion of articles with authors who had clinical and/or training COIs that omitted to disclose these roles as COIs in the manuscript, (c) the proportion of articles with authors who had clinical and/or training COIs that erroneously declared in the manuscript that the authors had no COIs, and (d) whether COI omissions were in violation of journal policies.

We selected studies from behavioral journals, as opposed to examining all autism ABA intervention studies, for three reasons. First, the bulk of autism ABA intervention studies are published in journals devoted to behavioral research. Second, publishing policies and practices are cultivated at the journal level, as journal editors and publishers are responsible for setting and enforcing policies. Third, conclusions that can be drawn in regards to publishing practices in specific journals may be more useful for proposing action steps that can be taken by individual editors and publishers.

We chose to focus on clinical and consultative COIs, as opposed to examining all potential COIs, for four reasons. First, these COIs present clear financial stakes, as the researcher’s employment is dependent on clients and practitioners perceiving ABA as an efficacious method for supporting autistic people. The financial incentives for other COIs, such as when researchers are board members but not paid staff members for an entity that provides intervention services, may be less clear. Second, ABA researchers who are employed in these clinical and/or consultative positions can use their published research as advertisements for the efficacy of their services, enhancing the financial incentives for positive findings. Third, these COIs are often directly stated in journal submission policies as the types of COIs that must be disclosed, and researchers across disciplines generally agree that these roles constitute COIs. Finally, clinical and consultative COIs are easy to locate in comparison to other COIs (e.g., the receipt of speaker fees, or royalties received from book sales), because clinical providers and training consultants often advertise their services via web pages. The relative ease of locating these COIs allows for a more accurate estimation of their prevalence, in comparison to other COIs that may not be possible to find via web searches and are not routinely disclosed.

## Methods

### Journals

We examined eight journals with a main focus on disseminating research on behavioral interventions, including: *Behavior Modification*, the *Journal of Applied Behavior Analysis, Behavior Analysis in Practice, Perspectives on Behavior Science*, *Journal of the Experimental Analysis of Behavior*, *Journal of Behavioral Education*, *The Analysis of Verbal Behavior*, and *The Psychological Record*. We searched each journal website for policies related to COI disclosures, and report this information in Table 1.

**TABLE 1:** Author Submission Guidelines Relevant to Conflict of Interest Disclosures.

Journal	COPE member	Conflict of Interest Policy from Author Submission Guidelines
*Behavior Modification*	Yes	No policy on journal website
*Journal of Applied Behavior Analysis*	No	No policy on journal website
*Behavior Analysis in Practice/Journal of Behavioral Education/The Analysis of Verbal Behavior/The Psychological Record*	Yes	Authors are requested to disclose interests *that are directly or indirectly related to the work submitted for publication*. Interests within the last 3 years of beginning the work (conducting the research and preparing the work for submission) should be reported. Interests outside the 3-year time frame must be disclosed if they could reasonably be perceived as influencing the submitted work. Disclosure of interests provides a complete and transparent process and helps readers form their own judgments of potential bias. This is not meant to imply that a financial relationship with an organization that sponsored the research or compensation received for consultancy work is inappropriate …
		**Employment:** Recent (while engaged in the research project), present or anticipated employment by any organization that may gain or lose financially through publication of this manuscript. This includes multiple affiliations (if applicable) …
		**Non-financial interests:** In addition, authors are requested to disclose interests that go beyond financial interests that could impart bias on the work submitted for publication such as professional interests, personal relationships or personal beliefs (amongst others). Examples include, but are not limited to: position on editorial board, advisory board or board of directors or other type of management relationships; writing and/or consulting for educational purposes; expert witness; mentoring relations; and so forth
*Perspectives on Behavior Science*	Yes	All authors are requested to include information regarding sources of funding, financial or non-financial interests, study-specific approval by the appropriate ethics committee for research involving humans and/or animals, informed consent if the research involved human participants, and a statement on welfare of animals if the research involved animals (as appropriate)
*Journal of the Experimental Analysis of Behavior*	No	No policy on journal website

*COPE = Committee on Publication Ethics.*

### Article Selection and Coding

We reviewed articles from the eight journals listed above that were published over a 1 year period (September 2019–September 2020), starting with the most recently available issue and backtracking through issues until a full year was covered. First, titles and abstracts were scanned to determine if the study examined an intervention strategy, reviewed a set of intervention strategies, or provided evaluative commentary on intervention strategies. Next, the participant information in the full text was reviewed to determine if at least one autistic participant was included in the study, or, if the article was a review/commentary, to determine if autistic participants were included in at least one of the primary studies that were included in the paper.

If a study was selected for inclusion, author names were recorded, and a Google search was conducted to determine if the author was employed in a clinical practice providing ABA services, provided private ABA services, or served in a training/consultancy capacity to ABA providers (university faculty who taught courses in BCBA programs were not considered to have this COI). If we could not locate evidence that a given member of the research team held a clinical/consultative COI at the time the study was conducted, we coded this as “no COIs.” As such, our COI counts are likely underestimates of the true number of researcher COIs. The KB-B and SC overlapped on 20% of articles to determine inter-coder agreement on designating an article as having at least one author with this COI, which was 86%. Finally, each full text article was scanned to determine if there was a COI disclosure statement. If such a statement was located, it was copied verbatim onto the coding spreadsheet, and a determination was made as to whether the statement covered the clinic and/or consultative COI identified in the first coding step. Because there were so few statements disclosing COIs, coding determinations were made by consensus between the KB-B and SC.

## Results

From the eight journals we examined, 180 articles met our inclusion criteria. Only five studies used group designs; the remaining 175 studies were either SCDs, reviews that included SCDs as primary literature, or commentaries on interventions/procedures that incorporated SCD research as evidence. Of the 180 included studies, 151 were authored by at least one person with a clinical and/or training consultancy COI (84%). A total of 501 unique author names were searched, and 260 were found to have a clinical and/or training consultancy COI (52%). COI statements were absent in 105 studies (58%), 70 studies included statements declaring no authors held COIs (39%), and only five studies included statements declaring COIs (3%). Of the 70 studies that declared no COIs, 61 of these were found to have at least one author who provided ABA clinical services and/or training consultations to ABA providers (87%). Two of the five studies that declared COIs disclosed the receipt of royalties from book sales, but did not mention relevant training consultancies performed by the author. Therefore, only 2% of studies adequately accounted for clinical/training consultancy COIs. Information regarding COIs and COI disclosures by journal are presented in Table 2.

**TABLE 2:** Conflict of Interest Information by Journal.

Journal (Most Recently Available Impact Factor)	Total articles	Articles with clinical/training COIs	COI statements	COI statements declaring no COIs	COI statements inaccurately declaring no author COIs*
Behavior Analysis in Practice (NA)	39	34 (87%)	36	32	27 (84%)
Behavior Modification (2.105)	15	15 (100%)	15	15	15 (100%)
Journal of Applied Behavior Analysis (2.108)	87	70 (80%)	0	NA	NA
Journal of Behavioral Education (.894)	18	14 (78%)	15	14	11 (79%)
Journal of the Experimental Analysis of Behavior (1.616)	9	8 (89%)	0	NA	NA
Perspectives on Behavior Science (1.219)	4	3 (75%)	1	1	1 (100%)
The Analysis of Verbal Behavior (NA)	7	7 (100%)	7	7	7 (100%)
The Psychological Record (1.010)	1	0 (0%)	1	1	0 (0%)

COI = Conflict of Interest, NA = Not Available or Not Applicable.

*Percentage calculated from total number of COI statements declaring no COIs.

Authors with COIs were located in seven of the eight journals; only one journal (*The Psychological Record*) did not have any authors with clinical/training consultancy COIs, but this journal contributed only one article relevant to our analysis. For the remaining seven journals, 75–100% of articles were authored by researchers with clinical/training consultancy COIs. In five of the six journals with articles providing COI statements, 79–100% of these statements falsely declared no COIs. The sixth journal only contributed one article to this analysis, and it was not authored by researchers with COIs.

Five of the eight journals provided instructions for authors regarding the disclosure of COIs, which, in our interpretation, included requirements to disclose clinical and/or training roles (readers are again referred to Table 1). For four of the five journals with false statements regarding COIs, these statements were in violation of journal policy regarding COI disclosures. For two of three journals that did not provide COI policies (the *Journal of Applied Behavior Analysis* and the *Journal of the Experimental Analysis of Behavior*), there were no COI statements included in any of the articles reviewed. For the third journal (*Behavior Modification*), all 15 articles selected for inclusion provided identical COI statements: “The author(s) declared no potential conflicts of interest with respect to the research, authorship, and/or publication of this article.” Despite these statements, all 15 articles were coded as having at least one author with a clinical and/or training consultancy COI. It is unclear how COIs were defined for these authors in the submission instructions, or at what point in the submission process they were prompted to provide this statement. Six of the eight journals are COPE members, and five of the six (all but *The Psychological Record*) published articles falsely claiming that authors did not have COIs.

## Discussion

Our findings corroborate ethical concerns raised by autistic people, autistic researchers, and non-autistic researchers in regards to ABA autism intervention research. In the articles we examined, nearly all authors who were employed in clinical and training consultancy roles either omitted to declare them as COIs in their published reports (i.e., there was no COI statement provided), or falsely claimed that the authors held no COIs. In many instances, such statements were in clear violation of the journal’s submission guidelines. In our process of searching for these COIs, we found particularly egregious cases where researchers posted links or reference to their published research on websites advertising their private clinical/training consultancy services. As such, these individuals are using their research to market their clinical expertise to prospective clients, but still claiming that their research is free of COIs.

The reasons for such a high prevalence of clinical/training consultancy COIs are likely linked to how ABA researchers are trained to conduct research. Many BCBA graduate programs simultaneously provide training in clinical practice and research methods, which means the majority of program graduates hold dual roles as ABA researchers and practicing BCBAs. Further, more established researchers may be considered leading experts in clinical practice, allowing for the possibility of branding themselves as consultants to existing BCBA practitioners. Researchers may gain important insights into intervention strategies via hands-on clinical practice and consultation roles that can positively inform their research; however, the financial incentives associated with such roles also present clear COIs that should be readily disclosed in research reports. Our findings indicate that ABA researchers not only maintain their BCBA credentials, they hold active roles as clinical providers or training consultants. There was variation in the specific roles held by researchers deemed to have COIs in this category. Roles could include employment as clinicians in large regional ABA centers or private ABA clinics, employment as CEOs/directors of ABA clinics and training consultancies, and employment as clinician, director, or training consultant in University-based clinics.

The prevalence of COIs, and the failure to disclose them, is an issue for autism intervention research more generally, and is not specific to ABA journals (Bottema-Beutel et al., 2020). However, while we recognize that the current study is not directly comparable to Bottema-Beutel et al. (2020) examination of COIs in group-design intervention literature, it is worth noting that they found COIs in 70% of reports when considering *all* COI types, as compared to the current study in which COIs were found in 84% of reports when restricting our search to exclusively cover clinical/consultancy COIs. Omitting to disclose clinical/consultancy COIs, or declaring that no COIs exist when they in fact do, could be common practice precisely because this COI is so prevalent and not considered particularly noteworthy. In addition, there are so few examples of COI statements in this literature that disclosure is simply not a norm governing researcher conduct. Because ABA autism intervention research is routinely published in ABA-specific journals, submitted reports are likely peer-reviewed by researchers with the same COIs as the submitting authors, and handled by editors who also have these COIs. This insular publication process has culminated in the production of a vast body of literature that has not adhered to basic ethical standards in regards to COI disclosures. The result of this failure of oversight, at a minimum, is that the extent to which we can be confident in study findings is greatly reduced.

Conflicts of interests that involve the provision of ABA services to autistic people or private consultation to ABA providers are pervasive in autism ABA intervention literature. Additionally, the failure to clearly disclose these roles as COIs is equally pervasive. COIs have the potential to introduce bias into the research process in ways that are not always known to the researcher, and ethical guidelines stipulate that COIs should be disclosed in published reports so that appropriate scrutiny and skepticism can be applied to research findings. Reviews that have assessed the quality of ABA research provide evidence that the majority of these studies – both for group design and SCD research – are designed in such a way that the risk of bias is high (Davis et al., 2019; Bottema-Beutel and Crowley, 2020; Sandbank et al., 2020). COIs such as those described in this paper may provide insight into why poor study quality has persisted (Dawson and Fletcher-Watson, 2020).

### Limitations

This study is limited by the fact that it covers only 1 year of publication, is restricted to only one type of COI, and focuses exclusively on journals devoted to publishing ABA research. Additional research may determine if trends in COI disclosures change over time, if the prevalence of COI reporting across different types of COIs, and if COI reporting differs for ABA studies that are published in ABA journals as compared to journals that publish a variety of intervention types.

### Recommendations and Implications

For ABA journals that do not have a prominently displayed COI disclosure policy (or do not have a policy at all), we recommend journal editors clearly indicate the necessity of declaring clinical/training consultancy roles as COIs, and feature these policies prominently in their instructions for authors. The COIs we explore in this paper already appear in most COI disclosure policies (including several of the journals included in this study), but there may be additional “grey area” COIs with risks of bias that are less clear and more difficult to protect against. A consensus-led tool for identifying and properly disclosing actual, potential, and perceived COIs could be developed and disseminated by journal editors, which benefit researchers both within and outside ABA autism intervention research. We also recommend much stronger oversight so that submitting authors actually follow the policies in place, and in cases where there are violations, editors have a responsibility to investigate and publish corrections as necessary.

Applied behavior analysis researchers, in turn, should routinely and clearly state any ABA clinical or training consultancy roles they perform when submitting research reports. Universities that prepare BCBAs and simultaneously provide research training should include information about ethics related to COIs in their curricula, so that ABA researchers are aware of these issues from the beginning of their careers (Dawson and Fletcher-Watson, 2020). We also recommend that ABA researchers develop procedures that reduce the risk of bias that these COIs likely introduce. In group design intervention research, intervention developers (who have a related, but different COI) have outlined protocols in which they remain a member of the research team, but partition themselves from the collection and analysis of data (Eisner et al., 2015). Likewise, interventionists who are on the research team should remain separate and independent from data collection and analysis teams. Data collection and analysis team members should be recruited who do not have a vested interested in the intervention being provided and are able to remain naïve to whether a child is in an intervention or control phase. Finally, analysis plans should be pre-registered prior to the launch of the study (including plans to protect against bias due to COIs. All procedures designed to reduce bias due to COIs can then be included in the method section of the published report, which would include any deviations from pre-registered procedures. These suggestions may serve as a useful starting point, but we believe it would be helpful for researchers to develop more formal guidance (similar to the Consolidated Standards for Reporting Trials checklist; Schulz et al., 2010) that all researchers could reference and follow, and journal editors could enforce as part of their publication standards.

Given that clinical/training consultancy COIs are so prevalent among ABA autism intervention researchers, implementing these reforms may require a significant restructuring of how research in this area is conducted. This restructuring is necessary if autistic people, and other stakeholders including parents of autistic children, researchers, and practitioners (we note that many of these latter three categories are also autistic people) – are to have any trust in the veracity of ABA autism intervention research findings. While mistrust in ABA research and practice goes beyond the prevalence of COIs, considerable improvements in COI disclosures and increased protections against their influence on research findings, is a small but necessary step toward improving stakeholder perceptions.

## Data Availability Statement

The raw data supporting the conclusions of this article will be made available by the authors, without undue reservation.

## Author Contributions

KB-B conceptualized the project, co-designed the study, participated in data collection, analyzed the collected data, contributed to interpreting the analysis, and drafted the manuscript. SC co-designed the study, participated in data collection, contributed to interpreting the analysis, and edited the manuscript. Both authors contributed to the article and approved the submitted version.

## Conflict of Interest

KB-B has previously received fees for consulting with school districts on intervention practices for autistic children and teaches courses on topics including applied behavior analysis. After the completion of this manuscript (but prior to its acceptance), KB-B accepted speaker fees in the amount of $750 to discuss her work on conflicts of interest. She also receives royalties for a co-edited book entitled Clinical Guide to Early Interventions for Children with Autism, published by Springer. At the time of publication, the total amount of royalties received for this work was $261. SC was formerly affiliated with an entity that trained students to become Board Certified Behavior Analysts and provided early Intensive Behavioral Intervention.
